# Neuroplasticity and Motor Rehabilitation in Multiple Sclerosis

**DOI:** 10.3389/fneur.2015.00059

**Published:** 2015-03-18

**Authors:** Ilona Lipp, Valentina Tomassini

**Affiliations:** ^1^Institute of Psychological Medicine and Clinical Neurosciences, Cardiff University School of Medicine, Cardiff, UK; ^2^Cardiff University Brain Research Imaging Centre, School of Psychology, Cardiff University, Cardiff, UK; ^3^IRCCS Fondazione Santa Lucia, Rome, Italy

**Keywords:** brain plasticity, motor recovery, rehabilitation, multiple sclerosis, MRI

## Introduction

Motor symptoms are common and disabling across the phases and forms of multiple sclerosis (MS). Disease modifying treatments help to prevent their development, but most of their management is through rehabilitation. Current rehabilitation approaches are based on physical therapy tailored to the individual’s needs (1). The efficacy of these approaches, however, is limited, as it is purely based on clinical grounds, and is largely unpredictable in the individual case, where several factors, including location, extent, and severity of MS damage, can contribute to individual variation in rehabilitation outcomes (2–7). Therefore, an improved understanding of the neural processes underlying functional recovery and driven by rehabilitation, as well as the development of novel recovery interventions that fully exploit the individual patient’s potential to recover motor function remain a clinical necessity and a research priority (8).

## Neuroplasticity Underpins Recovery of Motor Function in MS

Plasticity is the ability of the nervous system to adapt to the ever-changing conditions of the environment, encountered during development and learning (9–11). Within the central nervous system, such plasticity is sustained by a variety of changes in gray matter (e.g., neurogenesis, synaptogenesis, changes in neuronal morphology), in white matter (e.g., changes in the number of axons, axonal diameter, fiber density, axonal branching and trajectories, myelination), and in other tissue compartments (e.g., glial cell size and number, angiogenesis) (12).

Experimental and clinical studies suggest that brain plasticity also occurs in disease (13), where adaptation to damage contributes to the preservation or to the recovery of function (14, 15). In MS, the bulk of evidence suggests that plasticity limits the clinical impact of damage, by establishing patterns of brain activity different from those of healthy volunteers, and accompanies improvements in motor performance with practice, by adaptively reorganizing those altered patterns (6). Indeed, studies on spontaneous recovery after a MS relapse show that changes in activation patterns occur with the resolution of active inflammation (16–18) and parallel recovery of motor function (16, 18). Recovery-oriented interventions can also drive these changes further by reorganizing or restoring altered patterns of brain activity (19) and improving behavior even at higher levels of disability and damage (5). Such interventions may also induce clinically meaningful changes in brain structures (20–23), possibly as a result of activity-dependent remyelination.

Not all of the changes in brain activity occurring in MS are adaptive and thus behaviorally beneficial. Evidence suggests that plasticity can also be maladaptive and thus contribute to or sustain disability (24, 25). Indeed, maladaptation may help to explain the functional differences that are observed between clinical stages and forms of MS (26), beyond individual variation in adaptive plasticity and structural reserve. Evidence of maladaptation calls into question, the increase in MS damage as the only factor that limits functional reorganization, as maladaptation itself can contribute to incomplete recovery and progression (27). Probing the limits of plasticity is challenging in MS because of the widespread and multifaceted nature of the disease, with the involvement of both gray and white matter (28), within (29), and outside (30) MS lesions, in the brain as well as in the spinal cord (31). The combination of neurophysiological methods and network-approach to data analysis can offer ways to probe the brain plastic reserve (6) and its behavioral consequences (32). Future interventional studies that interfere with cortical function or studies that assess concurrent structural changes may also disambiguate the relative contributions of inefficient versus insufficient versus ineffectual plasticity (6).

## The Exploitation of Neuroplasticity Promotes and Enhances Rehabilitation-Driven Motor Recovery

To promote the individual’s potential for recovery in MS by exploiting adaptive plasticity, we need to test novel recovery interventions that combine a strong biological rationale with monitoring of clinically meaningful functional and structural brain reorganization. For these studies, the methodology and neuroscientific rationale need to be carefully considered.

*Methodologically*, optimized trials that use enriched designs to manipulate behavior through interventions would offer a novel experimental framework for testing efficiently the promotion of adaptive plasticity. Markers of recovery that combine clinical and neurophysiological measures could provide insight into the clinically meaningful mechanisms of plasticity and offer a tool for early detection of effects of intervention. Markers predictive of recovery could improve stratification of patients in clinical trials, while developing a personalized approach to recovery-oriented interventions. Technology, especially in the field of neuroimaging [e.g., high field magnetic resonance imaging (MRI)], novel measurements, and sophisticated network-level analysis (33, 34) can now meet this increasing demand for novel markers and predictors. The development of computer-based behavioral measurements also offers sensitive and objective ways to target even subtle deficits and quantify behavioral improvements (35).

*Neuroscientifically*, an improved knowledge of changes in the brain that accompany functional recovery remains crucial, with the need to distinguish truly adaptive versus maladaptive changes (24), and changes representing compensation versus those representing restitution (36). Additionally, the development of novel strategies for motor recovery requires an improved understanding of the properties of the normal motor system, such as its flexibility and the stability of induced functional and anatomical changes, which vary with development (37) and previous experiences (38, 39) and thus inevitably influence the plastic response to damage (13). Approaches that adopt pharmacological and/or non-pharmacological modulation of neuroplasticity to enhance functional recovery represent promising strategies (6). While they pose methodological challenges in terms of prediction of response, qualification of markers of recovery, and development of appropriate outcome measures, these approaches hold promise for clinically meaningful benefits (6) and open therapeutic opportunities for more disabled cohorts (40, 41). Combining experimental evidence with clinical studies will offer a scientifically grounded rationale to develop novel interventions that may predispose (42), promote (5, 19), or enhance (6) plasticity underlying functional recovery. In this regard, future therapeutic approaches with novel disease modifying treatments hold promise for combined preventative and neuroprotective (43) or restorative (44) effects that increase further the prospects of and scope for functional recovery.

## Conclusion

Rehabilitation of motor function is a major component of MS management that is supported by neuroplasticity, i.e., the brain’s ability to adapt to MS damage or disability. Developing novel and more effective rehabilitation approaches, therefore, requires an improved understanding of brain plasticity that can be exploited in recovery interventions. The need for novel rehabilitation approaches, underpinned by promoted and enhanced neuroplasticity, challenges traditional experimental designs. This challenge can be addressed using methodological advances, especially in neuroimaging, which allow improved understanding of mechanisms and detection of intervention effects. In this article, we provide a critical overview of the current knowledge of neuroplasticity and its modulation in MS motor rehabilitation and we offer a vision for future directions of research in this field.

## Conflict of Interest Statement

Dr. Ilona Lipp is funded by the MS Society UK. Dr.Valentina Tomassini has received grants for studies on functional recovery in MS from the MS Society UK, MS Society Italy, MS International Federation, Italian Ministry of Health, Merck Serono, Switzerland.
